# Transforming of the Tumor Microenvironment: Implications for TGF-*β* Inhibition in the Context of Immune-Checkpoint Therapy

**DOI:** 10.1155/2018/9732939

**Published:** 2018-12-02

**Authors:** Stefanie Löffek

**Affiliations:** Skin Cancer Unit of the Dermatology Department, West German Cancer Center, University Hospital Essen, University Duisburg-Essen, and the German Cancer Consortium (DKTK), 45147 Essen, Germany

## Abstract

Significant breakthroughs have been achieved in the fields of oncogenic signaling inhibition and particularly immune-checkpoint blockade has triggered substantial enthusiasm during the last decade. Antibody-mediated blockade of negative immune-checkpoint molecules (e.g., PD-1/PD-L1, CTLA-4) has been shown to achieve profound responses in several of solid cancers. Unfortunately, these responses only occur in a subset of patients or, after initial therapy response, these tumors eventually relapse. Thus, elucidating the determinants of intrinsic or therapy-induced resistance is the key to improve outcomes and developing new treatment strategies. Several cytokines and growth factors are involved in the tight regulation of either antitumor immunity or immunosuppressive tumor-promoting inflammation within the tumor microenvironment (TME), of which transforming growth factor beta (TGF-*β*) is of particular importance. This review will therefore summarize the recent progress that has been made in the understanding of how TGF-*β* blockade may have the capacity to enhance efficacy of immune-checkpoint therapy which presents a rational strategy to sustain the antitumor inflammatory response to improve response rates in tumor patients. Finally, I will conclude with a comprehensive summary of clinical trials in which TGF-*β* blockade revealed therapeutic benefit for patients by counteracting tumor relapses.

## 1. The Blockade of Immune-Checkpoints in Cancer Therapy

A common feature uniting all tumor entities is the ability of these cells to escape cytotoxic destruction by the immune system. Therefore, the use of novel checkpoint inhibition immunotherapy belongs to the most promising approaches as it aims to increase antitumor immunity. Under physiological conditions, immune-checkpoints are important to maintain a certain self-tolerance in order to prevent self-destruction [1, 2]. However, in the context of cancer therapy, blocking the immune-checkpoints CTLA-4 (cytotoxic T-lymphocyte-associated antigen 4) and/or PD-1/PD-L1 (programmed death 1 receptor and PD-1 receptor ligand 1) significantly facilitates immune cell activation, thereby generating durable clinical responses in cancer patients [3, 4]. For example, a large body of evidence has established that tumor cells, particularly malignant melanoma, elevate their PD-L1 expression in order to transmit inhibitory signals in several immune effector cells via binding to their PD-1 receptor. Thus, interfering PD-1/PD-L1 ligation with monoclonal antibodies is a promising tool to increase endogenous antitumor activity [5–8]. In addition, the ligation of B7-1 and B7-2, which are specifically expressed on antigen-presenting cells (APCs), to their receptor CTLA-4 prevents T cell activation. Clinical trials using antibodies that block the ligation of PD-1 (nivolumab, pembrolizumab) or CTLA-4 (ipilimumab), with their ligands, revealed them to be efficient in more than 15 cancer types, including metastatic melanoma, bladder carcinoma, non-small cell lung cancer, and breast and renal cell carcinoma [9–12]. However, despite this unexampled success, either many patients completely fail to respond, or even more concerning, among the initial responding patients, tumor regression often occurs, suggesting that tumor cells acquire therapy-induced resistance.

## 2. Multiple Mechanisms of Therapy Evasion: Intrinsic/Primary versus Therapy-Induced Resistance

Despite the overall success of innovative cancer therapies, including targeted and immune-checkpoint therapies, resistance mechanisms still represent a major clinical problem. Thus, tumors that show initial response can rapidly become therapy-tolerant and progress. Unfortunately, drug resistance has turned out to be a multifactorial phenomenon, which is especially based on the property of tumor cells to present high phenotypic cell plasticity and heterogeneity [13, 14]. Therefore, fundamental efforts are being made to gain insights into the mechanisms that are either existent in intrinsic/primary resistance (patients do not respond to checkpoint inhibition) or leading to therapy-induced resistance (patients initially respond but eventually relapse) [15]. Apparently, it is very difficult to dissect the molecular mechanism particularly of therapy-induced resistance by using only clinical tissue samples, as this dynamic process requires tumor samples throughout the course of treatment. To this regard, a discriminatory model has been established that allows the accurate prediction of clinical response to anti-PD-1 therapy in melanoma patients [16]. Using 46 blinded samples from melanoma patients (before and during therapy) the authors could show that the spatiotemporal appearance of preexisting CD8(+) T cells at the invasive tumor margin and inside the tumor is the major predictor for the response to anti-PD-1 therapy, yet the underlying molecular mechanisms remain poorly understood. Two recent published studies aimed to unravel molecular basis of anti-PD-1 resistance. The first study verified that sufficient intratumoral T cell infiltration is a prerequisite for the response to anti-PD-1 therapy [17]. However, it is commonly accepted that the activity of tumor infiltrated lymphocytes can be inactivated by the TME through multiple pathways [18]. Interestingly, the treatment of immunotherapy-resistant Ag104Ld tumors with Ab-LIGHT (homologous to lymphotoxin)-based therapy can activate the lymphotoxin *β* receptor (LT*β*R) signaling specifically in tumor cells which is leading to enhanced secretion of multiple chemokines. These chemokines trigger massive T cell infiltration into the TME. As these newly recruited T lymphocytes are easier to be activated and less prone to be suppressed, targeting LIGHT might be a strategy to increase immune-checkpoint therapy [17].

The authors of the second study made use of a large scale genomic and transcriptomic analysis which revealed that patients resistant to anti-PD-1 therapy show an increased expression of gene sets that are associated with TGF-*β* signaling, epithelial to mesenchymal transduction (EMT), wound healing, angiogenesis, and increased monocyte and macrophage chemotaxis, indicating TGF-*β* as a key enforcer of immune tolerance and an obstacle in the optimal activation of the immune system during immune-checkpoint therapy [19]. Along that line, lack of response to anti-PD-L1 (atezolizumab) therapy in patients with metastatic urothelial cancer was associated with enhanced TGF-*β* signaling in adjacent fibroblasts within the TME and coinhibition of PD-L1 and TGF-*β* converted immune-desert mouse tumors to an inflamed phenotype, leading to tumor regression [20].

## 3. Clinical Relevance of TGF-*β* Signaling and EMT Markers in Tumor Diagnosis and Therapy

The current literature has brought ample and clear evidence for the interrelationship between epithelial-mesenchymal plasticity and oncogenic pathways especially in advanced melanoma [21–24]. Within the framework of this concept expression of distinct EMT marker has been proposed to serve as diagnostic marker, including the loss of membranous E-cadherin during the vertical growth-phase at deep sites in the dermis and in metastatic nodules and the* de novo* expression of vimentin as a predictor of hematogenous metastasis [25–27]. In patients with melanoma, renal cell, colon, and breast cancer, TGF-*β* plasma levels are elevated and correlate not only with tumor progression and the formation of metastases, but also with poorer clinical outcome [28–34]. Malignant cells often secrete large amounts of TGF-*β* whose autocrine and paracrine activities can increase the heterogeneity within a tumor subsequently leading to the ability of tumor-subpopulations to survive antitumor therapy, contributing to disease progression and seed metastasis [35]. In this context, TGF-*β* can act as a potent inducer of integrins and VEGF gene expression thereby promoting tumor cell dissemination and tumor-induced angiogenesis [36, 37]. Accordingly, therapeutically targeting TGF-*β* has been shown to prevent the development of melanoma bone metastases and decreased the progression of established osteolytic lesions in preclinical mouse models [38]. Silencing TGF-*β* expression in B16F0 melanomas reduces tumor growth and enhances antitumor immunity in C57BL/6 mice as well [37]. Furthermore, increased expression of immunosuppressive PD-L1 by tumor cells has been observed in mouse models of lung cancer. In this scenario PD-L1 expression was strongly regulated by miR-200/ZEB1 axis (the regulatory axis for EMT). As a consequence, infiltrated lymphocytes were less in number and mainly inactive, thus linking the mesenchymal phenotype of tumor cells to T cell exhaustion and tumor tolerance [5]. Another report highlights the importance of MED12, a negative regulator of TGF-*β* signaling, in the development of therapy resistance. Loss of MED12 promotes the transition of a mesenchymal phenotype, which is accompanied by chemotherapy resistance in colon cancer patients and resistance to the tyrosinase inhibitor gefitinib in lung cancer. Interestingly, the inhibition of TGF-*β* receptor-mediated signaling by the small molecule inhibitor galunisertib (LY2157299) restored drug responsiveness in MED12 deficient cells, suggesting that MED12-deficient tumors may benefit from anti-TGF-*β* therapy [39].

Lymphocyte priming by APCs, such as dendritic cells (DCs), within the sentinel lymph node is a prerequisite for the efficient generation of antitumor T cells. Worth mentioning, tumor-derived TGF-*β* is capable of inducing immunosuppressive conditions, not only within the TME but also in sentinel lymph nodes by the impairment of DCs and increased number of protumoral myeloid-derived suppressor cells (MDSCs) and regulatory T cells (Tregs) [40]. This work indicates that the systemic inhibition of TGF-*β* might improve both the priming and the effector phase of cytotoxic T cells.

In summary, these preclinical* in vitro* and* in vivo* studies implicate a clinical perspective for the inhibition of TGF-*β* signaling in cancer therapy.

## 4. TGF-*β* Signaling Pathway and Its Implication for Cancer Immunotherapy

### 4.1. TGF-*β* Signaling

The family of transforming growth factor-*β* comprises more than 40 members, including TGF-*β*, bone morphogenic proteins (BMP), activins, and related proteins, all of which are defined through their specific roles in multiple cellular functions, like cell proliferation, death, invasion, and differentiation, not only during development but also during pathogenesis of various diseases.

In mammals three TGF-*β* isoforms are described (TGF-*β*1, 2, and 3) which are the primary mediators of TGF-*β* cell transduction. All isoforms are synthesised as propeptides that form dimers which require maturation before being able to bind to their receptors. These dimers are composed of the C-terminal TGF-*β* ligand as well as an N-terminal latency-associated propeptide (LAP), with which the complex gets sequester to proteins of the extracellular matrix (ECM). Thus, the release of the bioactive cytokine from the ECM, e.g., during wound healing, inflammation, or cancer development and progression, mainly by matrix metalloproteinases (MMPs) or thrombospondin-1, rapidly increases TGF-*β* bioavailability and subsequently accelerated TGF-*β* signal transduction.

Once activated, TGF-*β* binds to the serine-threonine kinase receptor TGF-*β* receptor II (T*β*RII) which phosphorylates TGF-*β* receptor I (ALK5). In the absence of TGF-*β* both receptors exist as homodimers. Binding of TGF-*β* results in the formation of a tetrameric receptor complex that propagates the signal by phosphorylation to the intracellular downstream effector proteins SMAD2 and SMAD3 (SMAD2/3). Upon engagement of phosphorylated SMAD2/3 with SMAD4 this oligomeric complex is translocated into the nucleus. Finally, the cooperation with many sequence specific transcription factors results in context-dependent regulation of transcription. In order to abate excessive TGF-*β* signaling, the activation of SMAD2/3 is inhibited by SMAD6 and SMAD7 [47, 48]. It is increasingly apparent that additional signaling pathways further define the actual cell response to TGF-*β* and that T*β*R activation can transduce signals via the noncanonical arm of signaling pathway that engages, for example, Rho-like GTPases and MAPK signaling as well as the activation of PI3 kinases [49].

### 4.2. TGF-*β* Mediated Transformation of Immune Response in Cancer

Regarding cancer development and progression, TGF-*β* is a multifaced cytokine; while it appears to have antitumorigenic functions in early steps of neoplastic transformation it takes on protumorigenic functions in later stages [50]. The tumor suppressive effects of TGF-*β* in normal cells and even in early carcinomas include mainly the inhibition of cell proliferation, the enforcement of differentiation states, and the induction of apoptosis. Historically, the initial evidence for protumorigenic effects of TGF-*β* was based on its ability to induce a mesenchymal phenotype in epithelial tumors, a process which is commonly described as the epithelial to mesenchymal transition (EMT). However, during the last decades it became clear that TGF-*β* orchestrate the crosstalk of almost all cell types within the TME, including, for example, fibroblast and endothelial cells. Studies aiming to analyse this complex crosstalk within the TME revealed that accelerated TGF-*β* signaling during tumor progression induces tumor cell migration, invasion, and formation of distant metastasis. At this stage tumor cells can escape from TGF-*β*-mediated antiproliferative control, either by the activation of signals via the noncanonical arm of signaling pathway or by gaining somatic mutations in components of the TGF-*β* pathway [51].

Importantly, as the genetic ablation of* Tgfb1* in mice results in spontaneously activated T lymphocytes, severe postnatal inflammatory reactions associated with tissue damage, organ failure, and a rapid death of knockout mice, TGF-*β* appears as a master regulator of inflammatory processes too [52]. And indeed, as a pleiotropic cytokine TGF-*β* impacts basically every cell type of the adaptive and the innate immune system [53, 54]. As such, TGF-*β* has been shown to suppress T cell proliferation and regulates lymphocyte differentiation. For example, TGF-*β* stimulates the generation of regulatory T cells (Tregs), characterized by the expression of CD25 and the transcription factor Foxp3. Although Tregs are initially identified to prevent autoimmune disease it is meanwhile well accepted that within the TME this subpopulation facilitates immune tolerance [55, 56]. Accordingly, overexpression of TGF-*β* in CT26 colorectal carcinoma cells enhanced tumor growth by suppressing antitumor T lymphocyte response in immune competent Balb/c mice [40]. Although accumulation of Tregs was seen in primary tumors and metastatic lymph nodes in a mouse skin melanoma model, depletion of CD25(+) Foxp3(+) T cells in lymphatic organs did not delay melanoma development, indicating that Treg functions might be replaced by other immunosuppressive cells [57]. Considering the fact that especially melanoma cells secrete large amounts of MCP-1 (monocyte chemoattractant protein-1, also known as CCL-2) in response to TGF-*β*, enhanced recruitment of protumorigenic monocytes most likely contributes to immunosuppression [58]. Among this line of evidence, increased levels of mesenchymal marker and monocyte and macrophage chemotactic genes (CCL-2, CCL-7, CCL-8, and CCL-13) are associated with innate anti-PD-1 resistance, indicating TGF-*β* as a key enforcer of immune tolerance and an obstacle in the optimal activation of the immune system during immune-checkpoint therapy [19]. In contrast, favorable clinical response to anti-PD-1 therapy was, among others, associated with enhanced expression of granzyme B on tumor-infiltrated CD8(+) cytotoxic T cells (CTLs) [16]. To this regard it is of great importance that TGF-*β* acts on CTLs by inhibiting the expression of the predominant cytolytic gene products, perforin, granzyme A, granzyme B, Fas ligand, and interferon *γ*, which are collectively responsible for CTL-mediated tumor cytotoxicity [59].

Cells of the innate immune system are targets of TGF-*β* as well. Here, TGF-*β* exhibits antagonistic functions on antigen-presenting and phagocytic cells during the process of antigen-recognition and clearance, for example, by inducing protumorigenic M2 macrophage polarization and N2 neutrophils [60, 61]. The TGF-*β*-mediated inhibition of functional maturation of natural killer (NK) cells results in a high number of immature NK cells and eventually impaired recognition and clearance of tumor cells [62]. Furthermore, recent work has shown that impaired TGF-*β* signaling in DCs enhances their ability to present tumor antigens in order to activate the adaptive immune system [63].

### 4.3. Clinical Perspective: TGF-*β* Inhibition in Clinical Trials

As preclinical studies implicate the use of TGF-*β* inhibition as a potential therapeutic target, monoclonal antibodies against all three isoforms of TGF-*β*, as well as T*β*R inhibitors, are currently tested in various solid cancers (reports of all clinical trial summary results are published on http://www.clinicaltrials.gov). For example, the TGF-*β*2-targeting antisense molecule trabedersen (AP12009) has been assessed in phase I/II clinical trial in patients with advanced pancreatic or colorectal cancer and malignant melanoma. The molecule appeared to be safe and well tolerated, and enrolled melanoma patients showed improved median overall survival [41]. In a phase I/II study with patients with high-grade glioma trabedersen showed a significant survival benefit over standard chemotherapy [42]. Also, a phase I clinical trial with 29 patients with advanced malignant melanoma showed an increase of 11.1 weeks in the median progression-free survival (PFS) upon treatment with the monoclonal anti-TGF-*β* antibody fresolimumab (GC1008). The major drawback in this study was the development of reversible cutaneous keratoacanthomas/squamous-cell carcinomas and hyperkeratosis [43]. A phase I trial with the same antibody in combination with radiation therapy in metastatic breast cancer patients is currently ongoing and the estimated study completion date is June 2019 (NCT01401062). A phase 1b/2 study (NCT01373164) was performed in order to determine a safe and tolerable dose of galunisertib (LY2157299) in combination with the cytostatic drug gemcitabine in patients with solid malignancy. In this trial both compounds together had an acceptable safety/tolerability profile and the median PSF was 64 days, indicating minor efficacy in cancer patients with advanced or metastatic pancreatic cancer. Unfortunately, no CR or PR was achieved [44].

In addition to the ubiquitously expressed T*β*Rs (T*β*RII and ALK5), the activin receptor-like kinase-1 (ALK1) has more distinct expression properties as it is preferentially expressed on proliferating vascular endothelial cells; thus inhibition of ALK1 might block tumor-induced angiogenesis. Binding of the BMP9 and 10 results in intracellular signaling via the phosphorylation of SMAD1, 5, and 8. PF-03446962 (Pfizer) is a humanized monoclonal antibody against ALK1, which has been studied in several phase I/II studies in various solid tumors, including colorectal and bladder cancer. In patients with advanced hepatocellular carcinoma 12 patients (50%) achieved stable disease, which lasted ≥12 weeks in 7 patients. Unfortunately, no CR or PR was reported (NCT00557856) [45]. Even more concerning, an open-label phase III trial (NCT01006252) of tasisulam (Y573636, an ALK5 inhibitor)* vs.* chemotherapy was placed on hold after randomization of 336 melanoma patients when a safety review indicated an imbalance of possibly drug-related deaths in patients receiving tasisulam. Nevertheless, although this clinical trial was closed early due to toxicity, tasisulam was considered unlikely to be superior to the chemotherapy (paclitaxel) [46].

As the TGF-*β* biology might already suggest, all blocking strategies are faced with the issue that TGF-*β* signaling is involved in many normal physiological functions and its pleotropic nature implies serious complications when completely blocked* in vivo*. Nevertheless, some of the above-mentioned TGF-*β* transduction inhibitors revealed good safety profiles along with prolonged progression-free survival. In this regard it is worth mentioning that anti-TGF-*β* antibodies might suppress the transition from epithelial/differentiated tumor cells to mesenchymal/invasive tumor cells, thereby preventing the metastatic dissemination rather than killing the tumor. Therefore, combinatorial therapy with immune-checkpoint inhibition becomes a feasible therapeutic strategy.

### 4.4. Combinatorial Inhibition of Immune-Checkpoints and TGF-*β*

As the response to anti-PD-1 monotherapy appears to be mainly limited by the number of preexisting cytotoxic T cells, concurrent TGF-*β* inhibition provides a powerful strategy to (a) improve T cell priming within the lymph nodes, (b) enhance cytotoxic destruction of tumor cells, and (c) reduce the appearance of immune suppressive immune cells. Accordingly, Vanpouille-Box and coworkers were among the first authors that impressively showed that triplet regimens of synergistic combinations of conventional radiation, immunotherapy, and the inhibition of TGF-*β* lead to durable tumor regression in preclinical models of metastatic breast cancer [64]. They identified TGF-*β* as the major obstacle in the generation of effective CD8(+) T cell response during radiation therapy as the inhibition of TGF-*β* was leading to tumor-specific T cell mediated regression of irradiated tumors. However, despite the increase in immune cell infiltrate the majority of tumors did not undergo complete regression, due to an upregulation of PD-L1 and -2 in neoplastic and myeloid cells and PD-1 on intratumoral T cells. This additional immunosuppressive effect was consequently abolished in 75% of anti-TGF-*β*/anti-PD-1 treated irradiated tumors, suggesting that the inhibition of both PD-1 and TGF-*β* is a good strategy to improve the clinical outcome of patients undergoing radiation therapy. Along that line of evidence, dual inhibition of ALK5 kinase activity (using galunisertib) and PD-L1 (monoclonal antibody clone 178G7) resulted in complete tumor regressions in colon carcinoma mouse models (CT26). The tumor regression was associated with enhanced T cell activation signatures, demonstrating the potential synergy when cotargeting both pathways [65].

Most recently, two groups have independently shown that TME-associated TGF-*β* is the major driver of immune suppression resulting in the lack of therapy response, in both preclinical models of metastatic colorectal cancer (CRC) and metastatic urothelial cancer (UrC) [20, 66]. As TGF-*β* contributes to the development of malignant CRC and metastasis, the authors have made use of a quadruple-mutant mouse model (conditional loss of* Trp53*,* Apc, Tgfbr2*, and mutant* Kras*) that develops immune-desert intestinal tumors and liver metastasis. Here, the observed benefit of T*β*R inhibition in reducing liver metastasis required the cytotoxic activity of CD8(+) cells. Furthermore, dual blockade of T*β*R and PD-L1 promoted a complete regression of liver metastasis and prolonged the survival of CRC model mice [66]. Absence of lymphocytes can be observed in nearly 50% of bladder cancer as well, suggesting that only a small subset of patients benefit from immune-checkpoint therapy. Thus, a large cohort of patients with metastatic urothelial cancer has been analysed using transcriptome RNA sequencing. While elevation of TGF-*β* signal transduction genes was associated with lack of response to atezolizumab (anti-PD-L1) in bladder tumors with immune excluded phenotype, therapy response was associated with a gene signature of CD8(+) T effector cells [20]. Interestingly, only the therapeutic inhibition of both TGF-*β* and PD-L1 promoted complete tumor regression in EMT6 mouse mammary carcinoma, which was fully dependent on CD8(+) infiltration. Also, this study revealed that TGF-*β* signaling (monitored by pSMAD2 staining) was particularly elevated in nonimmune cells, indicating the contribution of stromal cells in immunotherapy [20].

It is however worth mentioning that there is a discrepancy between the presence of CD103(+) tumor infiltrating T cells (TILs) and the concept of blocking TGF-*β* signaling. Several groups have shown that CD103, also known as integrin *α*E, is expressed on a subset of CD8(+) TILs in multiple solid human tumors (including ovarian, lung, cervical, and head and neck cancer patients cancer as well as endometrial adenocarcinoma) and it is known that its expression is induced upon TCR engagement and exposure to TGF-*β*1 [67–72]. Interestingly, the presence of CD103(+) TILs has been shown to be a prognostic factor for prolonged survival and the concurrent high expression of checkpoint molecules implicates elevated response rates to immune-checkpoint inhibition.

In human papilloma virus (HPV)-induced cervical cancer CD103 has been identified as a biomarker for the rapid assessment of tumor-reactive T cell infiltration [70]. In this study the authors could show that the TME was rich in pSMAD2/3 expression, most likely induced by a direct activation of the TGF-*β*1 promoter by HPV-16 E6 and E7 oncoproteins. As this TGF-*β*-rich environment has been shown to be the key determinant of CD103 induction in T-cells the authors speculate that the same may therefore hold true for other types of HPV-mediated cancers, such as head and neck squamous cell carcinoma. With regard to the combined inhibition of immune-checkpoints and TGF-*β* it will therefore be interesting to assess whether response rates differ between HPV-positive and -negative cancers.

Nevertheless, the effort to combine TGF-*β* and immune-checkpoint inhibition in human clinical trials has already begun (see Table 1). An entirely innovative and exciting strategy to combine both targets was recently shown [73]. The investigators developed a first-in-class bifunctional fusion protein composed of a monoclonal antibody against PD-L1 fused to a TGF-*β* “trap” (T*β*RII fusion protein M7824). A phase 1, open-label, dose-escalation and dose-expansion trial of M7824 with 19 patients with heavily pretreated advanced solid tumors revealed a manageable safety profile, one cervical cancer patient with confirmed CR, two durable confirmed PR (pancreatic cancer and anal cancer), one near-PR cervical cancer patient, and two patients with prolonged stable disease (pancreatic cancer and carcinoid). As this preliminary evidence of therapeutically success is very encouraging the investigators are aiming to study multiple expansion cohorts in a range of tumor types, including colorectal cancer and non-small cell lung cancer.

In addition, there is an ongoing phase I/II study of galunisertib (LY2157299) in combination with the anti-PD-1 antibody nivolumab in participants with advanced refractory solid tumors and in recurrent or refractory non-small cell lung cancer or hepatocellular carcinoma (metastatic and/or unresectable; NCT02423343). The estimated study completion date is December 2019. Likewise, galunisertib in combination with the checkpoint inhibitor durvalumab is currently tested in pancreatic cancer patients and the estimated study completion date will be June 2019 (NCT02734160).

## 5. Conclusions and Future Perspectives

Despite the vast amount of knowledge on immune-checkpoint therapy that has been accumulated in the last four to five years, we have just started to understand the impact of the TME on therapy resistance mechanisms. As discussed in the previous sections, the knowledge on the importance of well-regulated TGF-*β* signaling led to the concept of TGF-*β* inhibition; however response rates in monotherapies using, for example, fresolimumab or galunisertib have been similarly modest [43, 44]. As a matter of fact, the pleotropic nature of TGF-*β* implies serious complications when completely blocked* in vivo,* because cells have the ability to recover from inhibition by engaging alternative signaling pathways to circumvent the inhibition. Furthermore, rather than killing tumor cells, TGF-*β* inhibition might prevent the metastatic dissemination by blocking EMT. Of conceptual significance are the recent research findings revealing that high stromal TGF-*β* activity at tumor margins hinders T cell infiltration, suggesting a key role of TGF-*β* in tumor immune evasion. Given the fact that the PD-1 and the TGF-*β* pathway encompass independent and complementary immunosuppressive function implicate, that dual inhibition might be a brilliant strategy to obtain durable control of tumor growth and metastatic dissemination. Indeed, combined therapies revealed promising results in preclinical mouse models; however their beneficial effects need to be carefully evaluated. Thus, the current clinical trials that seek to combine TGF-*β* blockage with immunotherapy will prove the concept of this strategy in the near future.

## Figures and Tables

**Table 1:** Selection of TGF-*β* pathway inhibitors for cancer therapy in clinical studies. Reports of all clinical trial summary results are published at CliniclaTrial.gov.

**Inhibitor**	**Clinical trial**	**Reference**
Trabedersen (AP12009)	phase I/II clinical trial; advanced pancreatic or colorectal cancer and malignant melanoma	[41]

Trabedersen	phase I/II study; high-grade glioma	[42]

Fresolimumab (GC1008) in combination with radiation therapy	phase I trial; metastatic breast cancer patients	NCT01401062

Fresolimumab	phase I trial; malignant melanoma or renal cell carcinoma	[43]
		NCT00356460

Galunisertib (LY2157299) in combination with gemcitabine	phase 1b/2 study; patients with solid malignancy	[44]
		NCT01373164

Galunisertib in combination with nivolumab	phase I/II study; advanced refractory solid tumors and in recurrent or refractory non-small cell lung cancer or hepatocellular carcinoma	NCT02423343

PF-03446962	phase I/II study; advanced hepatocellular carcinoma	[45]
		NCT00557856

Tasisulam	open-label phase III trial; melanoma	[46]
		NCT01006252

Galunisertib in combination with durvalumab	phase I study; pancreatic cancer patients	NCT02734160

## Abbreviations

TGF-*β*: Transforming growth factor beta
T*β*RII: TGF-*β* receptor II
ALK5: TGF-*β* receptor I
BMP: Bone morphogenic proteins
ALK1: Activin receptor-like kinase-1
EMT: Epithelial to mesenchymal transduction
TME: Tumor microenvironment
CTLA-4: Cytotoxic T-lymphocyte-associated antigen 4
PD-1/PD-L1: Programmed death 1 receptor and PD-1 receptor ligand 1
APCs: Antigen-presenting cells
DCs: Dendritic cells
MDSCs: Myeloid-derived suppressor cells
Tregs: Regulatory T cells
CTLs: Cytotoxic T cells
NK: Natural killer cells
MCP-1: Monocyte chemoattractant protein-1 (also known as CCL-2)
PFS: Progression-free survival
CR: Complete response
PR: Partial response
CRC: Colorectal cancer
UrC: Metastatic urothelial cancer.
